# What is the cost of integration? Evidence from an integrated health and agriculture project to improve nutrition outcomes in Western Kenya

**DOI:** 10.1093/heapol/czz083

**Published:** 2019-08-29

**Authors:** Carol E Levin, Julie L Self, Ellah Kedera, Moses Wamalwa, Jia Hu, Frederick Grant, Amy Webb Girard, Donald C Cole, Jan W Low

**Affiliations:** 1 Department of Global Health, University of Washington, NJB Box #359931, 325 Ninth Avenue, Seattle, WA, USA; 2 Rollins School of Public Health, Emory University, 1518 Clifton Road, Atlanta, GA, USA; 3 Path, Bungoma, Kenya; 4 International Potato Center, Bungoma, Kenya; 5 Dalla Lana School of Public Health, University of Toronto, 155 College St Room 500, Toronto, ON, Canada; 6 International Potato Center, Nairobi, Kenya

**Keywords:** Agriculture, costs, food-based strategies, health, integration, nutrition

## Abstract

Integrated nutrition and agricultural interventions have the potential to improve the efficiency and effectiveness of investments in food security and nutrition. This article aimed to estimate the costs of an integrated agriculture and health intervention (Mama SASHA) focused on the promotion of orange-fleshed sweet potato (OFSP) production and consumption in Western Kenya. Programme activities included nutrition education and distribution of vouchers for OFSP vines during antenatal care and postnatal care (PNC) visits. We used expenditures and activity-based costing to estimate the financial costs during programme implementation (2011–13). Cost data were collected from monthly expense reports and interviews with staff members from all implementing organizations. Financial costs totalled US$507 809 for the project period. Recruiting and retaining women over the duration of their pregnancy and postpartum period required significant resources. Mama SASHA reached 3281 pregnant women at a cost of US$155 per beneficiary. Including both pregnant women and infants who attended PNC services with their mothers, the cost was US$110 per beneficiary. Joint planning, co-ordination and training across sectors drove 27% of programme costs. This study found that the average cost per beneficiary to implement an integrated agriculture, health and nutrition programme was substantial. Planning and implementing less intensive integrated interventions may be possible, and economies of scale may reduce overall costs. Empirical estimates of costs by components are critical for future planning and scaling up of integrated programmes.

**Key Messages**

- This is one of the few studies to address the challenge of capturing the costs of interventions that work across sectors to improve health and nutrition.
- The cost per beneficiary for the integrated agriculture-nutrition-health intervention was US$155, when considering women only, and reduced to US$110, when also including infants who attended postnatal care services. These costs are comparable with other community-based interventions.
- The costliest component of the intervention was integration, which included monthly feedback meetings and other co-ordination and monitoring activities by all partners and implementing agents (27%), followed by administration and overhead (23%) and training (15%).
- Scenarios for lowering costs through economies of scale and integration into routine services should be further explored.

## Background

Agricultural and nutrition-sensitive interventions can complement health service and nutrition-specific interventions to improve child outcomes (Hawkes and Ruel, 2006; World Bank, 2008; Ruel *et al.*, 2018). However, evidence of the cost of scale-up is largely limited to nutrition-specific interventions (Horton *et al.*, 2010; Bhutta *et al.*, 2013), such as behaviour change communication, micronutrient supplementation, food fortification and integrated child health days or child health weeks (Fiedler *et al.*, 2007). There is little evidence of the costs and benefits of integrating nutrition-specific and nutrition-sensitive interventions across multiple sectors (Ruel, 2001; Girard *et al.*, 2012; Masset *et al.*, 2012; Black *et al.*, 2015). Cost evidence will be crucial to support investments to scale-up integrated nutrition interventions (Hawkes *et al.*, 2012; Ruel and Alderman, 2013; Ruel *et al.*, 2018). Lessons from the health sector indicate that there may be cost efficiency gains from integration, though integration may also lead to an increase in overall programme costs (Atun *et al.*, 2010; Sweeney *et al.*, 2012; Siapka *et al.*, 2014; Obure *et al.*, 2016).

The Mama SASHA project explicitly integrated agriculture and nutrition interventions into existing Ministry of Health (MOH) antenatal care (ANC) services, with the aim of maximizing the benefits of orange-fleshed sweet potato (OFSP) on the health status of mothers and children <2 years of age. A comprehensive evaluation strategy assessed impact, acceptability, feasibility and affordability of the integrated approach (Machira, 2013; Cole *et al.*, 2016). An impact assessment demonstrated that the intervention package led to increased ANC utilization, improved nutrition and health knowledge, greater diet diversity and more frequent consumption of vitamin A-rich foods (Levin *et al.*, 2016). In addition, results from a nested cohort study demonstrated that health service promotion of OFSP was a feasible strategy to measurably improve nutrition knowledge, vitamin A intakes and vitamin A status (Girard *et al.*, 2017a,b).

Donors, governments and implementers need evidence on the costs and benefits of integrated, multi-sector nutrition interventions to assess value for money and affordability, and to make financial projections for scaling up effective programmes. This article presents our approach to cost documentation, analysis and evidence on the costs and affordability of Mama SASHA. The specific objectives were: (1) to estimate the total implementation costs and cost per beneficiary of the Mama SASHA intervention and (2) to identify potential efficiencies for scaling up the intervention to other locations where OFSP holds promise.

### Project overview

The Sweetpotato Action for Security and Health in Africa (SASHA) was a 5-year multi-partner project designed to improve the food security and livelihoods of poor families in sub-Saharan Africa by exploiting the untapped potential of sweet potato.^1^ The agriculture-health linkages Mama SASHA project in the Western Province of Kenya was a component of the larger SASHA project. The Mama SASHA project was integrated into the USAID/Kenya AIDS, Population and Health Integrated Assistance Program (APHIA II and APHIAplus) and bundled OFSP promotion and production support with ANC and enhanced nutrition and infant and young child feeding education. The overall Mama SASHA project goal was to improve the health status of pregnant women and the nutritional status of children up to 2 years through an integrated OFSP and health service delivery strategy in Bungoma and Busia counties, Kenya.

### Implementation activities

The Mama SASHA intervention was conducted through a combination of health facility and community-based strategies targeting pregnant women. After an initial pilot in 2009, the implementation start-up activities (planning and training) began in late 2010 and recurrent implementation activities ran from March 2011 to December 2013. See Cole *et al.* (2016) for a description and timing of pilot and intervention implementation activities. Vouchers for OFSP vines were the pivotal design element, used to link community health workers (CHWs), nurses, secondary vine multipliers (VMs) and agriculture extension agents to work together to increase OFSP production and consumption and increase health service utilization. Vine vouchers were the driving incentive for mothers to attend ANC clinics and a key integrating mechanism. At the community level, CHWs recruited women into pregnant women clubs (PWCs), informing women that they would receive a pair of vouchers for OFSP vines upon attending ANC clinics serving their community. At the facility, nurses issued vouchers and provided counselling on nutrition and healthy eating for pregnant women and infants. In agricultural fields, VMs provided women with vines upon receipt of each pair of vouchers. In addition, trained agriculture extension officers (AEOs) followed up with agronomic advice and home visits to assess and discuss OFSP planting and crop management.

Mama SASHA implementing partners (nurses, CHWs, AEOs, CHWs and VMs) received training to support the pregnant women with nutrition counselling, accessing vines and offering OFSP production support. Co-ordination and monitoring activities were critical for integrating the Mama SASHA project into existing ANC and postnatal care (PNC) services and for ensuring a shared understanding and coherent implementation of activities. Integration activities included annual, quarterly and monthly feedback meetings, transportation and per diems for all meeting participants, and NGO labour to support integration.

## Methods

This cost analysis estimated incremental total financial and unit costs for start-up and recurrent activities related to delivering the Mama SASHA activities, described above, at health facilities already supported by the USAID APHIAplus project. We used a mix of top-down expenditure analysis and bottom-up activity-based costing to understand resource use and financial costs. The analysis followed existing methodological guidance for primary health care costing in low- and middle-income countries (Creese and Parker, 1994; Walker, 2001; Fiedler *et al.*, 2008). We collected data on costs from project documents, monthly expense reports and interviews with key staff from the implementing organizations. Our approach allowed us to consider three mutually exclusive ways to classify costs: by activity, by input and by start-up vs recurrent implementation.

### Activity cost categories

We conducted activity-based costing and allocated the costs of the Mama SASHA project to several broad categories: planning, training, materials development, or delivery of community, health and agricultural support services. Most activities were comprised of costs related to personnel, travel, per diem, supplies and equipment depreciation. We created a separate activity category called ‘administration’ to capture overhead and indirect costs. We interviewed staff across implementing organizations to understand the timing and frequency of activities and personnel time allocated to specific Mama SASHA activities.

This analysis recognized that the intervention built on existing health and agriculture capacity and infrastructure. The activity categories reflect additional activities that were not part of existing ANC and PNC services; therefore, the financial cost estimates do not include the value of shared personnel costs from the MOH and the Ministry of Agriculture (MOA), or the costs of community-based activities by established cadres of CHWs already supported by APHIA II and APHIAplus. For example, we included the costs associated with new NGO activities like managing the receipt and redemption of vouchers, supporting PWC, or initiating and supervising agricultural activities for establishing vine multiplication of new varieties of OFSP. The current analysis adopted a provider prospective, where the provider was a combination of the Kenyan government and NGO services based in Kenya.

### Input cost categories

We also classified costs based on input categories for personnel, agriculture supplies, agriculture equipment, other consumable supplies, other capital equipment, transportation, travel per diem, allowances, mixed inputs, vouchers and other costs (Table 1). We estimated indirect administrative (overhead) costs for both international non-profit partners based on standard percentages used by each organization. For all agricultural and other equipment or capital goods, we included financial depreciation equal to the value of the capital equipment divided by useful life years, where useful life years was 10 years for the international non-profit project vehicles and 5 years for all other capital equipment or vehicles procured by the NGOs and used by project implementers, such as irrigation equipment used by the VMs. All costs were collected in Kenyan local currency and converted to 2013 US$ using an average exchange rate of 84 Kenyan Shillings per US Dollar (Central Bank of Kenya, 2013).

**Table 1.** Activity and input cost categories for the Mama SASHA project

	Description
Cost categories by activity	
1. Planning	Planning activities for project implementation during the start-up period; includes NGO personnel, supplies and transport costs.
2. Training	Training health workers, agriculture extension agents, community health extension workers and CHWs for integrated agriculture, health and nutrition. Includes venue costs, NGO personnel, transport and per diem.
3. Development of materials	Development of new nutrition counselling cards and posters and pamphlets with agriculture, health and nutrition guidance.
4. Awareness-raising and sensitization	Annual stakeholder workshops and activities at community level or local government level: include venue costs, NGO personnel, transport and per diem.
5. Establish plots for VMs or demonstration	Resources and inputs to establish plots for VM farmers: include equipment depreciation, seeds, fertilizer and labour.
6. Assure adequate and continuous supply of vines	Resources and inputs to assure adequate and continuous production of vines: includes equipment depreciation, seeds, fertilizer and labour.
	Value of voucher reimbursement.
7. Improve knowledge and practices for producing OFSP	Includes refresher training costs and costs of updated materials
	Planning and conducting field days, personnel, travel and supply costs.
	Planning and maintaining demonstration plots, personnel and supply costs.
8. Assure adequate and continuous supply of roots to households	NGO and MOA personnel, agriculture supplies and irrigation equipment.
	NGO agricultural extension personnel and transport costs to provide direct technical support to project beneficiaries.
	MOA agriculture extension personnel and transport costs to provide support to project beneficiaries.
	International NGO personnel and transport costs to support agriculture activities.
9. Meetings to co-ordinate and monitor integration of activities	Participation in all partner meetings: include annual, quarterly and monthly feedback meetings. Supplies transport, per diems and share of international and local NGO personnel costs.
10. Capital investment	Investments that last longer than 1 year. Depreciation included.
11. Administration	Overhead and indirect costs
12. Health intervention	Facility- and community-level visits and meetings to introduce the Mama SASHA vouchers for vines, providing improved nutrition counselling cards into existing antenatal and postnatal services, support to pregnant women’s clubs at the community level and establish and support recording procedures and monitoring; include Health NGO personnel, supplies and transport costs.
Cost categories by inputs	
1. Personnel	Value of personnel time.
2. Agriculture supplies	Seeds, fertilizer, pesticides, spray bottles, small tool, fencing material, posts, supplies for marking fields and varieties of sweet potatoes.
3. Other supplies	Stationary and other miscellaneous office supplies.
4. Agriculture equipment	Water tanks, irrigation equipment
5. Transportation	Fuel and maintenance of cars or motorcycles.
6. Overhead	Overhead costs were estimated for international NGOs based on standard rates used by each organization.
7. Contracted services	Maintenance of plots, logistical support.
8. Travel/per diem/allowances	Travel costs, including per diems and safari allowances
9. Other	Training registration fees.
10. Mixed inputs	Workshops or events where it was difficult to pull out specific input costs.

### Start-up and recurrent implementation costs

Start-up costs included costs on activities for planning, education and communication (IEC) materials development (December 2009 to December 2010), training programme development, establishing secondary VM plots, and promotion and awareness-raising activities (September 2010 to February 2011). Start-up costs were considered a type of fixed or capital cost, where we estimated their useful life years as 5 years (i.e. the life of the project). Recurrent costs were for inputs associated with maintaining the supply and demand for vines, supervision and support for health facilities, and integration activities, including any refresher training, occurring from March 2011 through December 2013.

### Omitted costs

All research-related costs were excluded from this analysis. In addition to implementing the Mama SASHA project described here, some implementing organizations had additional research costs to support the overarching SASHA project research objectives. Personnel, transportation and travel costs were proportionately allocated to research based on staff time spent between research and implementation activities. Only personnel time spent on Mama SASHA implementation was included.

### Data management and analysis

We transcribed the international and local NGO project expense reports into an excel template. All data were entered by organization and assigned activity and input codes based on the cost categories described above and in Table 1. The sum of the activity cost categories is comprehensive and includes all incremental financial costs to implement the integrated interventions.

### Project outputs

The cost analysis uses two main summary measures of overall intervention service provision: (1) the number of beneficiaries reached and (2) the total points of contacts with beneficiaries. We estimated both using monthly data collected as part of routine Mama SASHA monitoring (for details, see Cole *et al.*, 2016). Health service delivery indicators included the number of PWCs and the number of women per club session, ANC delivery tracking, PNC delivery tracking, and ANC monthly reporting on women receiving vouchers. On the agriculture side, indicators included voucher tracking, frequency of home visits for OFSP production and monitoring AEO visits. Monthly data were compiled and aggregated over the 3-year project period, by health facility, to estimate the total number of single-count women who received and redeemed vouchers, the number of women participating in PWC, and the number of voucher pairs issued during ANC and redeemed through VMs. See Supplementary Appendix Table SA1 for more details.

The number of project beneficiaries reached was defined as: (1) the total number of single-count women who received and redeemed vouchers; and (2) 1+ the number of infants who attended a PNC visit with their mother.^2^ The total number of contacts summarized the total number of times beneficiaries came into contact with implementing agents to promote the production and consumption of OFSP. Beneficiaries could participate in monthly PWCs held at the community level. They could interact with ANC or PNC health workers up to five times throughout their pregnancy and the newborn period. In addition, beneficiaries could receive at the ANC clinic four pairs of vouchers, which they could subsequently redeem for vines from participating VM farmers.

### Costs

We aggregated incremental financial costs by year and by NGO organization for the implementation phase (2011–13). Unit costs were estimated as incremental financial costs per service delivery output described above (Supplementary Appendix Table SA1). The cost per (direct) beneficiary was estimated as the total financial costs divided by the number of single-count mothers who have received and redeemed the voucher (or as total financial costs divided by the single-count mothers plus infants). The cost per contact was estimated as the total financial costs divided by the total number of contacts as defined above. We also estimated unit costs for critical intermediate outputs, such as the cost per pregnant woman’s club and the cost per voucher pair issued (Supplementary Appendix Table SA2). Lastly, we estimated resource allocation by activity and input, showing the breakdown of these costs as a share of total costs.

### Sensitivity and scenario analysis

We conducted sensitivity analysis to characterize uncertainty around cost estimates. Given that it was difficult to allocate shared programme costs for vehicles and transportation that supported concurrent implementation and research, we chose a high scenario estimate assuming a 25% increase in international NGO transportation costs. To estimate a more typical scenario for the intervention in the event that the MOH and MOA decided to continue supporting these activities beyond the life of the Mama SASHA project, the low scenario cost estimate applied a local staff salary to both the overall co-ordination and supervision role and the local agronomist’s position, as well as reduced indirect costs for international non-profit organizations.

## Results

### Project outputs

Over the 2011–13 project period, 5480 women participated in 215 pregnant women’s clubs. ANC nurses issued 7159 vouchers to 4605 women (Supplementary Appendix Table SA1). Although women were eligible to receive up to four pairs of vouchers throughout their pregnancy and postpartum period, many received, accepted or redeemed fewer than the total possible. Only 62% of the vouchers were redeemed. This was often because women received a sufficient quantity of vines for their land from one or more voucher pairs during previous visits. In some cases, women obtained vouchers during a dry period when they could not plant, given that most relied on rain-fed agriculture. Monitoring records showed that 4464 voucher pairs were redeemed by a total of 3281 women beneficiaries. Records also indicated that 1331 infants attended a PNC visit with mothers who received a voucher. Including both mothers and infants, the total number of beneficiaries was 4612. There were 17 103 points of contact with mother beneficiaries and 18 434 when including infants (Table 2).

**Table 2.** Summary of beneficiaries and total points of contact by health facility (2011–13)

Health facility	Mothers only		Including infants^a^	
	Beneficiaries	Total points of contact	Beneficiaries	Total points of contact
HF1 (health dispensary)	444	2071	601	2228
HF2 (health dispensary)	817	3699	1176	4058
HF3 (sub-district hospital)	1339	7981	1960	8602
HF4 (health centre)	681	3352	875	3546
Total	3281	17 103	4612	18 434

a Included infants of mothers who attended a PNC visit and received a voucher as part of Mama SASHA. Mothers may have attended other PNC visits with their infants that are not recorded here.

### Total and unit costs for women beneficiaries

Incremental financial costs are presented for the intervention in Table 3. These are presented as overall estimates and disaggregated by project timing, activity type and input type. Incremental financial costs and unit costs by the organization are presented in Table 4 (greater detail in Supplementary Appendix Table SA2). Total costs were estimated to be US$507 809 for the period from 2011 to 2013. Start-up costs accounted for US$50 165 (9.9%) of total project costs, and recurrent costs accounted for US$457 644 (90.1%). The cost per direct beneficiary was US$155. The cost, which reflected repeated interaction with beneficiaries via facility visits, pregnant women’s clubs and interactions with VMs, was US$30 per contact. Table 3 also shows the variation in costs between the two Kenyan agricultural NGOs and the international partners (INTL HEALTH and INTL AGRIC).

**Table 3.** Total incremental financial costs by cost category

Cost category	Total frontline costs (US$)	Total costs (%)
By implementation phase		
Start-up^a^	50 165	10
Recurrent	457 644	90
By organization type		
Local NGO 1	72 503	14
Local NGO 2	77 259	15
International Health	195 098	38
International Agriculture	162 949	32
By activity		
Planning	16 881	3
Training	73 545	14
Development of materials	12 442	2
Awareness-raising and sensitization	2399	0.5
Establish plots for VMs or demonstration	44 885	9
Improve knowledge and practices for OFSP	30 750	6
Assure adequate and continuous supply of roots to households	35 867	7
Health intervention	25 555	5
Meetings to co-ordinate and monitor integration of activities	136 222	27
Capital investment	12 231	2
Administration	117 030	23
By input		
Personnel	181 124	36
Agriculture supplies	11 910	2
Agriculture Equipment	5602	1
Vouchers	4915	1
Contracted Services	6325	1
Transportation (fuel and maintenance)	27 231	5
Travel/per diem/allowances	119 169	23
Other supplies	19 033	4
Mixed inputs	24 488	5
Overhead	100 252	20
Other	7761	2
Total	507 809	100

a Start-up activities included planning, training, development of materials, awareness-raising and sensitization and establishing plots for VMs. Refresher training is treated as a recurrent cost.

**Table 4.** Total incremental financial costs and unit costs by organization for the period 2011–13 (US$)

Organization^a^	Total financial costs (US$)	Cost per beneficiary (US$)	Cost per contact (US$)
Ag NGO 1^b^	72 503	57	13
Ag NGO 2^c^	77 259	38	7
INTL HEALTH	195 098	59	11
INTL AGRIC	162 949	50	10
Total	507 809	155	30

a Unit costs for Ag NGO 1 and Ag NGO 2 included costs and outputs specific to those organizations and the health facilities they supported. Unit costs for both international partners included costs for each organization and outputs from all facilities.

b Ag NGO 1 served women attending health facilities HF1 and HF2 in Table 2.

c Ag NGO 2 served women attending health facilities HF3 and HF4 in Table 2.

Each local agricultural NGO spent over US$70 000 during the 3-year project period. Local NGO #1’s unit costs were US$57 per beneficiary and US$13 per contact, whereas local NGO #2’s were US$38 per beneficiary and US$7 per contact. The differences in the unit costs reflect the location of the local NGOs and the number of women participating from the associated health facilities. NGO #2 supported more women from larger and more accessible health facilities (HF3 and HF4 in Table 2) that had a higher volume of monthly ANC clients, whereas NGO 1 worked with a lower volume of monthly clients from smaller and more remote health facilities (HF1 and HF2).

### Cost profiles

**Figure 1.**
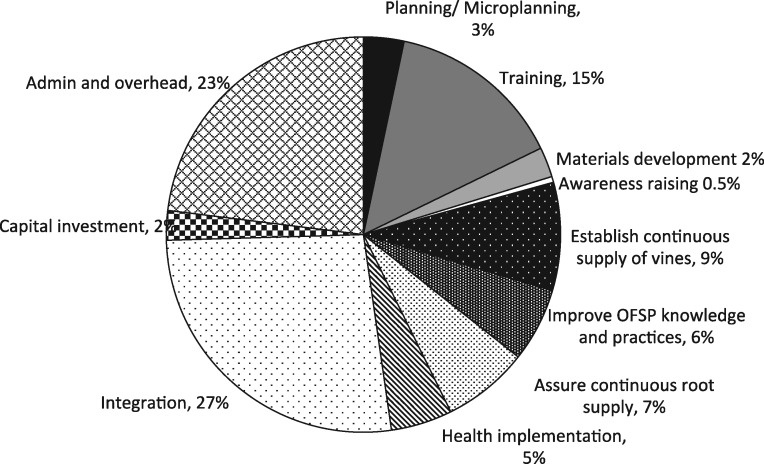
Cost profile by activity categories.

Three activities accounted for most of the expenses over the course of the project: integration, which included monthly feedback meetings and other co-ordination and monitoring activities by all partners and implementing agents (27%), administration and overhead (23%) and training (15%). An additional one-fifth (22%) of the total costs were used to support agricultural activities related to establishing a continuous supply of vines (9%), ensuring adequate and continuous supply of roots to households (7%) and improving knowledge and practices of OFSP among beneficiaries and the community (6%), while a relatively smaller share of financial costs went to supporting health facilities in vine voucher programme distribution (5%). The remaining budget (9%) was allocated to planning, development of training and ICT materials, awareness-raising and sensitization, and depreciation of capital investments (Figure 1).

**Figure 2.**
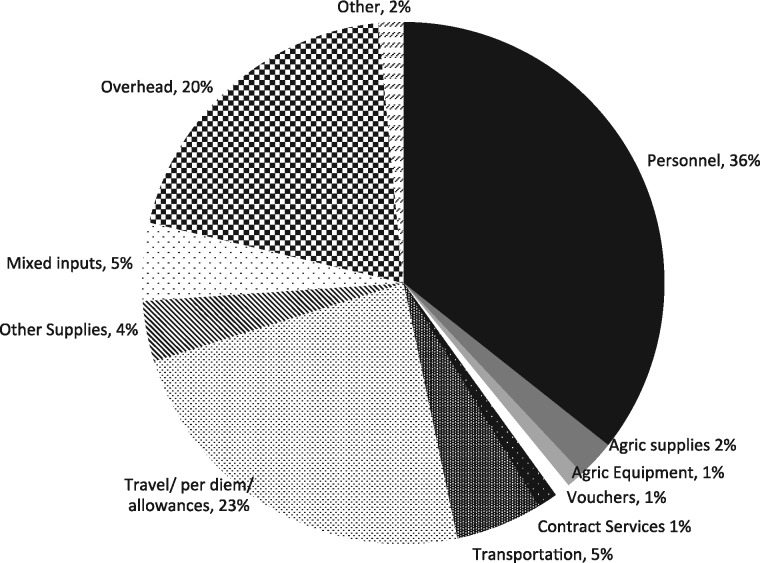
Cost profile by input categories.

Figure 2 presents the cost profile by input. Personnel costs accounted for 36% of total costs by the international and local NGOs, followed by transport and per diem costs (23%) and overhead (20%). The total value of voucher reimbursement was around US$5000, which accounted for only 1% of total costs. Note that personnel costs were incremental financial expenditures for the Mama SASHA project, and did not include the staff time of nurses, health workers and agriculture extension agents, whose salaries were already being paid out of existing government funds. However, information obtained during operational research and key informant interviews indicated that approximately 6–7% of health workers and agriculture extension agents’ time annually was engaged with recurrent Mama SASHA activities (see Supplementary Appendix Table SA4).

**Figure 3.**
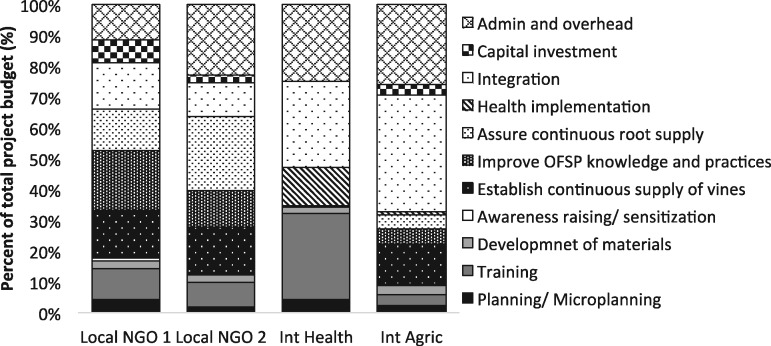
Cost shares by programme activity, by organization 2011–13 (%).

Figure 3 provides financial cost shares by the organization, reflecting the differing roles and responsibilities across each of the four organizations. As expected, the two local NGO costs were related to supporting agricultural activities to establish OFSP vines and assure their continuous supply and to improve OFSP production knowledge and practices. Although the international agriculture non-profit organization had some expenses related to supporting agriculture, the largest share of their costs was for project administration, overhead and supporting the integration of the agriculture and health components through stakeholder meetings and activities. Similarly, the international health non-profit organization had a large share of costs related to administration and overhead, but the largest share was for training.

**Figure 4.**
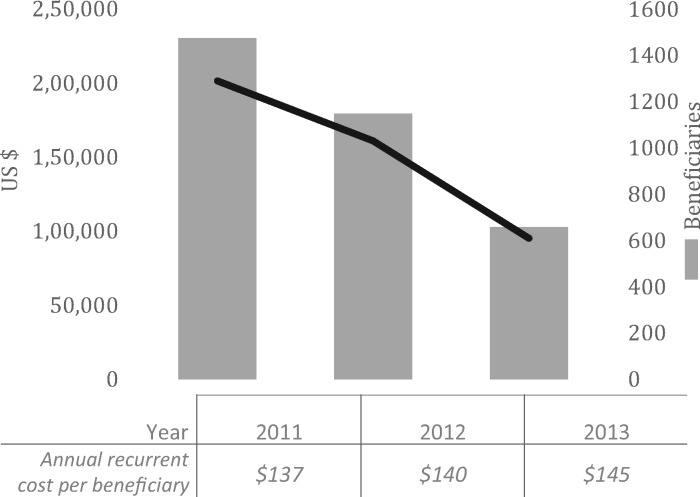
Annual implementation costs, beneficiaries and cost per beneficiary by year.

Total recurrent costs and output declined over the 3-year period (Figure 4). The project reached the greatest number of women in the first 2 years, resulting in an estimated average recurrent cost per beneficiary of US$137 in 2011, when participation was highest. By 2013, with declining participation, the cost was US$145 per beneficiary.

### Sensitivity and scenario analysis

Table 5 presents the low and high estimates compared with the baseline scenario laid out in Table 3. Increasing the international implementing organizations’ transportation and travel costs by 25% had the effect of increasing the cost per beneficiary by 4.5%, to $162 per beneficiary. In the low estimate scenario, where we replaced the expatriate salary and benefits with a local salary and benefits, the cost was reduced to $125 per beneficiary, a 19% decrease. When including infant beneficiaries, the cost per beneficiary ranged from $89 to $115. The cost per contact ranged from $24 to $31, or $22 to $29 when including infant beneficiaries (see Supplementary Appendix Table SA5 for details).

**Table 5.** Sensitivity analysis: total incremental financial costs and unit costs

		Women only		Including infants	
	Total cost (US$)	Cost per beneficiary (US$)	Cost per contact (US$)	Cost per beneficiary (US$)	Cost per contact (US$)
Low estimate	410 155	125	24	89	22
Medium estimate	507 809	155	30	110	28
High estimate	530 596	162	31	115	29

## Discussion

To our knowledge, this is the first study in sub-Saharan Africa to empirically estimate the costs of an integrated agriculture and health intervention implemented at both the community and facility levels. We estimated that the financial costs of reaching nearly 6000 beneficiaries (and over 18 000 contacts) in a 3-year period were just over US$500 000 or approximately US$170 000 per year, including start-up costs. When considering mother–baby pairs, the average unit costs were US$110 per beneficiary and US$28 per contact. Vine vouchers were the project’s critical integrating element: over 7000 pairs of vouchers were distributed to over 4600 women and infants. Each voucher and each mother receiving one or more vouchers were tracked from the health facility to the field. The financial value of the vouchers was low (US$1 per voucher pair redeemed), so the total reimbursement costs of vouchers comprised only a fraction of the overall project expenditure (1%). However, when considering more comprehensive costs of establishing a continuous supply of vines and the requisite health implementation activities for distributing and tracking the vouchers, the cost share of the voucher component was more substantial (around 20%).

This study estimated intervention costs in several ways in order to understand each sector’s separate and joint financial contributions to an integrated agriculture and health intervention. Inter-sector co-ordination and feedback mechanisms supported demand generation and ensured a sustainable supply of vines but significantly increased the cost per beneficiary. Early operational research evaluating the pilot component (PATH, 2011) highlighted the importance of integration, especially to ensure that the supply of vines could meet the demand from pregnant women. During project implementation, monthly feedback meetings, joint training and stakeholder meetings and refresher training workshops proved critical to successfully reaching women with consistent messages. Integration across actors and sectors and the establishment of a strong relationship between the health system and the community all required resources and financial support (over 25% of the total costs).

Previous studies have presented costs for nutrition interventions with similar complexities in linking health and community-based activities, such as treatment of severely malnourished children, and introducing OFSP through agriculture extension with enhanced nutrition education (Baker and Talukdar, 1996; Wilford *et al.*, 2012; Puett *et al.*, 2013, 2014; de Brauw *et al.*, 2018). For example, the Reaching and Engaging End User (REU) project in Eastern and Southern Africa developed OFSP vine distribution systems, provided an extension to farm households and nutritional knowledge to women in these same households. Like Mama SASHA, national agricultural and international NGOs carried out implementation. Unlike Mama SASHA, the REU project did not link to public health services. In Mozambique and Uganda, the average cost per adopting beneficiary (children aged 6–59 months and their mothers) was US$146 and US$132, respectively (De Brauw *et al.*, 2018).

Community-based management of severe acute malnutrition (CMAM) shares several similarities with the Mama SASHA project, though CMAM does not include an agricultural component. In Bangladesh, Puett *et al.* (2013) estimated that community-based management cost US$165 per child treated. One main difference between the treatment of severely malnourished children and an intervention targeted to pregnant women is the potential for economies of scale when working with pregnant women, since there are fewer severely malnourished children relative to the number of pregnant women in the majority of settings. In a similar study in Malawi, Wilford *et al.* (2012) found that CMAM cost $214 per child treated. As one might expect, given differences in focus and scope of these interventions, the Mama SASHA cost estimates fall between those of the REU project and CMAM estimates.

Other household food-based strategies have varied implementation costs. Unpublished reports by Helen Keller International provided cost estimates for a 1996 pilot of homestead food production programme promoting household gardening of vitamin A-rich foods in Bangladesh at an adjusted cost of 2013 US$68 per household garden (Baker and Talukdar, 1996). More recently, Puett *et al.* (2014) estimated the total financial costs (excluding household labour) of establishing and maintaining low-input vegetable gardens among households of people living with HIV in Zimbabwe to be 2010 US$1890 per household. These are higher than other reported food-based strategies because they capture the full implementation costs, including delays due to political unrest and price inflation due to economic crises.

### Caveats

Several study limitations are worth noting. First, this analysis presented information on financial costs and did not estimate the opportunity cost of time for health workers, agriculture extension agents, community volunteers and beneficiaries. However, key informant data presented in Supplementary Appendix SA5 suggested that this was small for paid workers. We captured all relevant financial implementation costs, using our judgement and assumptions to extract what we considered ‘project-related expenses’ and excluding research costs and project management costs related to the broader SASHA project.

Second, in parsing out non-project-related expenses, we tried to make consistent decisions on what to include and exclude but were challenged by different financial accounting methods across the four implementing organizations. NGOs coded or categorized inputs or activities differently for many expenses related to workshops, training, personnel, travel, transportation and indirect/overhead expenses. In particular, we had to use our judgement to allocate vehicles and transportation costs to research and implementation activities, since we did not have detailed information on how vehicles and transport supported project activities. We may, therefore, have underestimated implementation costs. Alternatively, we may have overestimated costs as vehicle and transport costs sometimes served both purposes.

Third, international NGOs incurred indirect costs related to fringe benefits and housing allowances for expatriate staff beyond the scope of the Mama SASHA project, so we estimated and included only the portion of those indirect costs that were directly related to the Mama SASHA project implementation. Further, we did not include labour and travel costs of international technical collaborators that contributed to the design of the proof-of-project concept and to annual work planning meetings, as the majority of these costs were for research design and management not directly related to intervention service delivery. Our results from the sensitivity analysis indicated that our cost estimates are fairly robust to changes in transportation, salaries of personnel and indirect expenses.

Fourth, we did not include the costs of implementing and evaluating the 2010 pilot programme, though it was instrumental in refining the programme, helping implementation to run more smoothly, and achieving higher uptake. Including the pilot would increase the overall project costs, though it would also increase the total number of beneficiaries reached by 2453 pregnant and lactating women. Finally, our cost per beneficiary does not capture the total number of infants and family members who consumed OFSP as an indirect result of Mama SASHA. Doing so would yield lower unit costs.

### Affordability and scalability

We estimated the Mama SASHA project to cost in the range of US$110 to $155 per direct beneficiary. Whether or not Mama SASHA is considered affordable will depend upon: the priorities of ministries of health and agriculture and international donors; sectoral organizations willingness to fund activities which cross sectors; the level of effort required to sustain or increase uptake over time; and the effects of moving to scale. At scale, we might observe economies of scope, as integrating activities allowed healthcare workers to more effectively reach their target population, leading to secondary cost savings (PATH, 2013) as well as opportunities to reduce implementation costs. Based on the Mama SASHA experience, nearby counties took the Mama SASHA concept to greater scale (Muoki and Agili, 2015). From 2014 to 2018, a modified model (SUSTAIN) was implemented in 33 health facilities across five counties adjacent to those targeted in Mama SASHA. Modifications to lower costs included: (1) targeting all pregnant women, and all mothers bringing children to the PNC clinic for monitoring or treatment; (2) just one voucher, instead of two (for the two varieties issued); (3) use of public sector agriculture services in lieu of agricultural NGOs; and (4), after 1 year of monthly feedback meetings, reduction to quarterly meetings. Although no formal impact assessment was conducted, the modified programme reached 35 362 women attending either ANC or PNC clinics. With an annual budget of US$480 000, or approximately $2 000 000 for the intervention period, a simple calculation estimates a cost of $57 per direct beneficiary reached with vines and nutrition counselling.

Future work might consider innovative ways to harness market forces and the private sector. In Western Kenya, there was a strong demand for OFSP vines by NGOs and community-based projects to improve agriculture, health and nutrition (P Muoki, personal communication, 9 January 2018). As a result of Mama SASHA, a dozen VMs established sustainable businesses to produce and sell surplus vines to other local NGOs. Previous studies have demonstrated the importance of early OFSP adopters and vine producers that can effectively lower the costs of extension services to neighbouring communities and encourage more rapid diffusion (De Brauw *et al.*, 2018).

## Conclusion

This study estimated the incremental costs of implementing an integrated nutrition and agricultural intervention in rural Kenya. Results suggest that the financial cost per beneficiary and the share of those costs needed for integrating activities across sectors can be substantial. Budgeting explicitly for joint training, regular monthly, quarterly and annual meetings will help future projects ensure that each sector has sufficient resources. Planning and implementing less intensive multi-sectoral interventions may be possible, and economies of scale may help to reduce overall costs. Empirical estimates of costs and their breakdown across sectors are critical to inform the design, planning and implementation of multi-sectoral programmes. Knowing costs by components enables thoughtful examination of where cost savings can be made with the goal of achieving the same level of impact.

## Ethical approval

All research protocols were approved by the institutional review boards at PATH and the Kenya Medical Research Institute.

## Supplementary Material

czz083_Supplementary_Appendix

## Footnotes

1 The project was led by the International Potato Center, with the nutrition/health component led by the International Health NGO PATH, together with to national NGOs focused on agriculture (CREADIS and ARDAP) and personnel from the MOH and the MOA.

2 We could only track the infants of Mama SASHA mother beneficiaries if they attended a PNC visit where the mother received a voucher for vines. This number underestimates the total number of infants born to the project’s beneficiaries.
