# Supplementary material for: What is the cost of integration? Evidence from an integrated health and agriculture project to improve nutrition outcomes in Western Kenya
Source: Health Policy Plan. 2019 Aug 29;34(9):646–55. doi: 10.1093/heapol/czz083 (PMC6880337; doi:10.1093/heapol/czz083)
Supplement: czz083_Supplementary_Appendix [file czz083_supplementary_appendix.docx]

Appendices

Appendix table 1. Service delivery outputs by facility (2011-2013)

Appendix table 2. Total and unit costs by organization for the period 2011-2013 (US$ 2013)

Appendix table 3. Cost summary by activity, input, frequency and year (US$ 2013)

**Appendix table 4. Estimated number of hours and percent of recurrent staff time for Mama SASHA activities by Ministry of Health and Ministry of Agriculture staff ^α^**

| **Implementing partner^β^** | **Annual number of hours** | | **Annual percentage of recurrent staff time (%)** |
| --- | --- | --- | --- |
| Health worker | 342 | 6% | |
| CHEW | 252 | N/A | |
| CHW | 3823 | N/A* | |
| AEO | 252 | 7% | |

**^α^** As part of operational research activities, we conducted key informant interviews with health workers, CHEW, CHW and AEOs on their time allocated to all Mama SASHA activities.  We calculated the total time per activity for weekly, monthly and one-time only annual events and then aggregated total time in minutes and days by year and for the overall project period (2011-2013).

**^β^** CHEW= Community health extension worker, CHW=community health worker, AEO=agricultural extension officer. CHEW and CHW are volunteer positions that do not have a set number of working days per year.

Appendix table 5. Sensitivity analysis for low, medium and high cost estimates (US$ 2013)
